# Moving urban political ecology beyond the ‘urbanization of nature’

**DOI:** 10.1177/0309132520903350

**Published:** 2020-02-24

**Authors:** Yannis Tzaninis, Tait Mandler, Maria Kaika, Roger Keil

**Affiliations:** 1234 University of Amsterdam, Netherlands; 1234 University of Amsterdam, Netherlands; 1234 University of Amsterdam, Netherlands; 7991 York University, Canada

**Keywords:** more-than-human, situated, suburbanization, urbanization of nature, urban political ecology

## Abstract

Urban political ecology (UPE) focuses on unsettling traditional understandings of ‘cities’ as ontological entities separate from ‘nature’ and on how the production of settlements is metabolically linked with flows of capital and more-than-human ecological processes. The contribution of this paper is to recalibrate UPE to new urban forms and processes of extended urbanization. This exploration goes against the reduction of what goes on outside of cities to processes that emanate unidirectionally from cities. Acknowledging UPE’s rich intellectual history and aiming to enrich rather than split the field, this paper identifies four emerging discourses that go beyond UPE’s original formulation.

[T]here can be no homelessness without an economic, political, and social process that produces ‘the home’ as a commodity; no refugees without practices of exile from a ‘country of origin’; no margin without a centre; no periphery without a core. (Kaika, 2004: 273)

The emancipatory potential of the urban planet lies in fact in the periphery. (Keil, 2018a: 1594)

## I Introduction: No outside left to conquer

In the opening scene of *Blade Runner 2049* (directed by Denis Villeneuve in 2017) we witness a dystopian future depicted against a monotonous synthesizer tune: vast, homogenized agricultural landscapes, dominated by synthetic farms and solar panels, constitute the future of Los Angeles’ extended periphery. The film depicts the ultimate state of capitalism’s environmental ills, ironically combining ecological collapse with renewable energy, free/slave labor and mass-produced synthetic food (Astley, 2018). In a fantastic extrapolation and inversion of the original, *Blade Runner 2049* moves our gaze away from the smoggy and rainy streets of a dystopian downtown LA to the horizontal planes of everywhere, a horror-scenario of a ‘continuous city’ sprawling over an ever-warming planet (Berger et al., 2017; Hern and Johal, 2018; Lerup, 2017). The extended urbanization of the planet is rendered full and final, with no possibility of escape to an alternative ‘outside’. *Blade Runner 2049* pictures an urbanization completed not only across but also beyond planet earth, where the outside and inside are no longer matters of concern; the only outsides left are ex-planetary dystopian/uninhabitable landscapes of waste and labor, those elements that Marx once thought of as the indispensable conditions of capitalist accumulation.

We may not be quite there yet, but the fires that burned in Alberta’s tar sands in 2016, across California in 2018–19 (Serna, 2019), and across Australia in 2019, bring into sharp relief the consequences of a violent ‘feral’ suburban development (Shields, 2012); development ‘where there shouldn’t be any’ (Arellano, 2018); development that has burned in the past only to be rebuilt with public blessings and even subsidies (Arellano, 2018); development that led to new waves of destruction. The juxtaposition of the original to the new cinematic *Blade Runner* landscapes acts as an analogy for the shifts in real landscapes of urbanization in less than one generation that produced the need for a recalibration of our analytical categories in urban geographies. While humans have become more urban in location and lifestyle, they have done so on exceedingly expansive terrain. In other words, whereas we now tend to live in urban environments, those urban environments are less dense than in the past and the more urban we get, the more suburban our existence appears (Angel et al., forthcoming). When Henri Lefebvre visited Southern California, around the time of the first *Blade Runner’s* release, he observed that Los Angeles presented ‘something stupendous and fascinating. You are and you are not in the city. You cross a series of mountains and you are still in the city, but you don’t know when you are entering it or leaving it. It stretches for 150 km, twelve million inhabitants. Such wealth! Such poverty!’ (Lefebvre, 1996: 208). Later, it became common, partly as a consequence of the Los Angeles School foray into the horizontalized region, to speak about the ‘Sixty-Mile Circle’ that circumscribes the urban (Soja, 1989: 224) in Southern California, or perhaps any city eventually. But that view was still from the center outward. It took another 30 years to understand that, while not all future cities will look like Los Angeles, they will certainly not follow the centralized Euro-US trajectories that Lefebvre as much as urban sociology and geography in the 20th century took as the model of development from which Los Angeles (or Houston, Johannesburg, Shenzhen, São Paulo or Djakarta) was considered an aberration.

During that same period (1980s–90s) critical urban geographical research and progressive urbanistic practice remained stubbornly focused on the urban center, even though it was expected that in the 21st century most of the world’s urban populations would live in the urban periphery. This focus was particularly pronounced in prescriptive and normative assumptions underlying policy and planning for urban sustainability (see for a critique Wachsmuth et al., 2016; Wachsmuth and Angelo, 2018).

The contribution of this paper is to recalibrate the project of UPE to these new urban forms and processes of extended urbanization that we have witnessed since the last quarter of the 20th century. We focus this exploration around the process of suburbanization as a fruitful way forward. Calling for an integrated political ecology of *sub*urbanization, we ask how and to what extent does the peripheral drive urbanization. And whether there is still a point in holding on to conventional uses of the terms ‘urban’ and ‘suburban’ altogether when it comes to exploring the urbanization of nature. This exploration responds to the call for resisting the reduction of what goes on outside cities to the dynamics and processes that emanate unidirectionally from cities (Keil, 2018a). Suburbanization here is defined as a function of what Lefebvre called extended urbanization (2003) (for an elaboration see Monte-Mor, 2014a, 2014b; see also Keil, 2018e; Simone, 2019), which includes all manner of processes of peripheral urbanization and has as a common denominator a combination of non-central population and economic growth with urban spatial expansion (Ekers et al., 2012: 407; Keil, 2018d: 11; McGee, 2011). Realizing the contentious debate around naming urban peripheries worldwide (Harris and Vorms, 2017), we choose suburbanization as the umbrella term used in a comprehensive fashion in critical studies in global suburbanisms for the last decade. Suburbanization in this sense includes a vast variety of expansions of form and process at the urban edge: informal settlements, gated communities, tower estates, kampungs, desakota, peri-urban villages and, yes, classical subdivisions of ground-related housing. The concept also entails suburban employment zones, office cities and aerotropolises, as well as recreational and infrastructural spaces.

More recently, the suburban lexicon has been moving to the acknowledgment of post-suburban forms which are characterized by densely layered dynamics of growth and decline, densification and de-densification, increasing demographic and economic diversity (Tzaninis, 2019) and contradictory socio-economic dynamics (Johnson et al., 2018; Lawton, 2019). Contributors to this critical suburban research program have gone beyond the common use of suburbanization and suburbanisms (as distinct suburban ways of life; see Moos and Walter-Joseph, 2017; Walks, 2013) in the US-centric tradition and have pushed towards critical scholarship on suburbanization that takes its origin in the periphery of cities outside the West (Keil, 2018d; Güney et al., 2019). This emerging suburban scholarship builds on traditions of conventional suburban scholarship in geography and other urban-related disciplines, for instance in historical geography (Harris, 2010); urban planning (Forsyth, 2012); demographic studies (for example the work of Champion [2001] on urbanization, surburbanization, counterurbanization and reurbanization); and classical political economy (Walker, 1981). The current critical suburban scholarship has focused on governance (Hamel and Keil, 2015), land (Harris and Lehrer, 2018) and infrastructure (Filion and Pulver, 2019). Large compendia of critical work have recently demonstrated the methodological variety of, contentious debates in, and global reach of these projects (Berger et al., 2017; Hanlon and Vicino, 2018).

The dynamics of uneven capitalist development at play in the forbidding worlds of both the fictional *Blade Runner 2049* and the present extended urban landscapes where the consequences of the climate crisis are being felt blur the boundaries of inside and outside, a classical definitory boundary constitutive of urban studies: the city is where countryside is not. In this situation, the dystopian present and future we face emphasizes further that the matter of concern should not be environments, or cities per se, but rather: ‘the urbanization *of* nature, i.e. the process through which all types of nature are socially mobilized, economically incorporated (commodified), and physically metabolized/transformed in order to support the urbanization process’ (Swyngedouw and Kaika, 2014: 462; emphasis in original). This interdependence between the ‘ecological’ and the ‘urban’ and its constitutive processes, along with the production of uneven geographies (Heynen, 2017), has been the focus of UPE for almost two decades (Connolly, 2018). As noted by Swyngedouw and Kaika above, a key characteristic of UPE scholarship is the development of an understanding of the ‘urban’ not as a bounded city within which political-ecological contestations are played out, but as a process of continuous socio-ecological transformation, a critical response to readings of urban and environmental issues that view ‘cities as purely social spaces…entirely separate from the countless non-human entities and organisms that are enrolled in, and help shape, urban life’ (Braun, 2005: 635).

Since its inception in the 1990s, UPE scholarship has been concerned with the examination of continuous socio-ecological transformations as a dialectic between inside and outside, urban core and periphery, local and global (Swyngedouw, 1996; Keil et al., 1998; Keil, 2003; Swyngedouw and Kaika, 2014; Keil and Macdonald, 2016). Examining the local in relation to the global, the margin in relation to the center, the unfamiliar as part of the familiar, the outside and the inside as one continuous process have been constitutive of the critical examination of the ‘urbanization of nature’ thesis (Kaika, 2004, 2005, 2014). Focusing on the geographies of the home, Kaika (2004) argued that the construction of a familiar safe ‘inside’ is predicated upon the simultaneous existence *and* exclusion of an unfamiliar ‘outside’. Whether undesirable environmental elements (disease, bad weather, refuse or sewage) or undesirable social elements (homelessness or refugees), the exclusion of the outside guarantees the familiarity of the inside. Being familiar in one’s home is dependent on being alienated, disconnected from social and natural processes that are supposed to take place outside this privileged core (Kaika, 2004, 2014). On a different scale but dealing with enclosure in the same logic, Marvin and Rutherford (2018) discuss ‘controlled environments’, namely urban spaces that are enclosed and engineered to create microclimates (p. 1144); they argue for a similar dichotomy of outside and inside, the former allowing for the construction of the latter so that it can protect from ‘turbulence and hostility’ (p. 1157).

As socio-environmental disasters like the wildfires of late demonstrate the relation between ecological problems and urbanization processes, and dominate political debates and agendas across scales, UPE’s call to overcome the distinction between inside and outside, to understand the dialectic between the local and the global that produces uneven development, to understand the core and the periphery as part of the same socio-environmental continuum is today more relevant than ever. A focus on the socio-environmental consequences of extensive urbanization is equally important politically. However, despite advanced theoretical debate within UPE and an increasing empirical focus on extended urbanization, an integrated research agenda for a UPE beyond the city has yet to be concretely developed. Indeed, it could be argued that UPE’s call to overcome the distinction between core and periphery, inside and outside, still privileges (at least discursively) the inside, the core, and the center as the spaces that dictate the logic of the outside, the periphery, the margin.

In this article, we shift the vantage point away from this privileged urban ‘core’ or ‘inside’, in order to sketch an integrated research agenda for a UPE beyond the city, by exploring if – and to what extent – it is also (or even mainly) the ‘margin’, the ‘outside’ and the ‘periphery’ that dictates the logic of the ‘core’, the ‘inside’. We argue that moving UPE beyond the city means taking seriously the dynamics of sub-urban, ex-urban or peri-urban spaces as representing ‘a meeting or overlapping of dynamics associated with the urban and the rural, a distinct and emergent landscape in-between’ (McKinnon et al., 2017: 354). Our call for a more-than-urban political ecology also responds to recent calls to situate UPE (Lawhon et al., 2014; Truelove, 2011; Loftus, 2012) and for increased attention on southern and subaltern urbanisms (Lawhon et al., 2014; Ranganathan, 2014; Roy, 2009; Silver, 2017; Truelove, 2016; Zimmer, 2010).

Following McKinnon et al. (2017), who note that the spaces and lives of those outside urban centers have been largely overlooked by urban geography, despite being part of the ‘urban’ population, we call for an integrated political ecology that examines processes and management practices beyond the privileged scales and places that have been the focal point of earlier UPE analysis. We suggest that this perspective has much to contribute in exploring thus far neglected actors and relations between institutions and political and economic forces involved in the urbanization of nature.

## II Moving urban political ecology beyond the ‘urbanization of nature’ thesis: Four theoretical challenges

In her review of UPE literature, Zimmer (2010) claims, first, that the definition of the city remains unclear and wonders ‘what characterizes the difference between city, peri-urban, and rural areas’ (p. 351). Regional dynamics after all have become especially crucial in understanding the patterns of urbanity (Neuman and Hull, 2009; Paasi et al., 2018). Second, she notes an under-acknowledged (semantic) tension between language such as ‘societal relationships with nature’ and Latour’s concept of hybridity, which rejects not only any distinction between ‘society’ and ‘nature’ but often discards both terms entirely. These challenges have been addressed and continue to be debated by UPE scholars over the last decade.

A series of more recent reports by Nik Heynen (2014, 2016, 2018b) and Collard et al. (2018) also reflect on the multiple directions UPE scholarship is heading towards. Using a chronological stage model, Heynen categorizes UPE scholarship in two ‘waves’. The ‘first wave’ of UPE, according to Heynen, includes foundational texts (*Concrete and Clay* (Gandy, 2002), *Social Power and the Urbanization of Water* (Swyngedouw, 2004), *Nature and the City* (Desfor and Keil, 2004), *City of Flows* (Kaika, 2005), *Lawn People* (Robbins, 2007)), and culminates with the 2006 volume *In the Nature of Cities*, edited by Heynen, Kaika and Swyngedouw. While a variety of approaches to, and applications of, UPE are present in this volume, most draw theoretical inspiration from Swyngedouw’s framing of metabolic circulation, reiterated in the second chapter (Swyngedouw, 2006). The ‘second wave’ of UPE, according to Heynen (2014, 2016, 2018b), comprises an emerging body of literature that is critical of UPE’s early framing. It includes research more attentive to race (Heynen, 2016), gender and sexuality (Heynen, 2018b), incorporating postcolonial, indigenous, feminist, and queer theory. While some authors maintain a commitment to a metabolic circulation framing, others move in new directions, often more concerned with the everyday and micro-politics.

Heynen’s chronological framing of UPE in two distinct waves may be useful for didactic purposes. However, his suggestion that UPE progresses in a somewhat linear manner with the latest scholarship being the ‘best’ and only ‘critical’ UPE scholarship is unhelpful. Suggesting that UPE scholarship is split into two camps (or waves) that somehow compete over which is the most ‘critical’ is an unfounded proposition, whose purpose in terms of enriching or moving the field forward is elusive. Therefore, in order to avoid inflicting unnecessary violence on sub-disciplinary histories, we propose instead to recognize the messiness of both earlier and recent UPE scholarship as a fruitful engagement amongst scholars, and to acknowledge the history of UPE as a heterodox field right from its inception. To suggest the contrary would mean editing out the complexities and critical engagement inherent in the field’s early debates and intellectual history (see also Connolly, 2018).^1^

Aiming to enrich rather than split the field, this paper identifies four emerging discourses in contemporary UPE, often in generative and productive dialogue with each other and with the diverse strands of recent and earlier scholarship. The first emerging discourse is a critique of UPE’s alleged methodological ‘city-ism’ and a call for UPE to ‘fulfill its Lefebvrian promises and contribute to a planetary, ecological, political understanding of contemporary urbanization’ (Angelo and Wachsmuth, 2014). The second emerging UPE discourse is the call for a ‘situated’ UPE coming from feminist UPE scholars and scholars working on and in the Global South who hope to create ‘the possibility for a broader range of urban experiences to inform theory on how urban environments are shaped, politicized and contested’ (Lawhon et al., 2014: 498). This work overlaps with theoretical and practical interventions ascribed to Southern urbanism and urban theories based on life in cities in the southern hemisphere (Bhan, 2019; McFarlane and Silver, 2017; Silver, 2014; Simone, 2004). The third emerging discourse tries to narrow the almost ontological rift between academic debate and policy/politics. Whilst academic debate is questioning ‘the urban’ not only as a valid conceptual framework but also as a distinct ontology, policy discourses put increasing emphasis on the urban and on cities as the object of inquiry, analysis, data collection and intervention. We argue that this rift between academic and policy debates has significant political as well as scholarly implications. The fourth emerging UPE discourse is a call to address the conceptual and methodological challenges around researching human and more-than-human actors by showing not only how ‘cities are produced through socio-natural metabolic flows originating “elsewhere”; but also how cities and their specific sociopolitical contexts and spatial configurations have strong implications for how…non-human natures are urbanized’ (Connolly, 2018: 2). In the following sub-sections – II(1), II(2), II(3) and II(4) – we explore further each one of these contemporary challenges for UPE scholarship.

### 1 Lefebvre’s planetary urbanization thesis and UPE

The planetary urbanization thesis (PU) has had a presence in theoretical and conceptual debates within UPE right from the beginning: ‘to speak now about UPE as central to urban studies in general may be interpreted as responding to Lefebvre’s challenge to create an urban science for an urban world’ (Keil, 2003: 728; see also Angelo and Wachsmuth, 2014; Soja and Kanai, 2007: 62). Perceptively, and compatibly with our argument, Castriota and Tonucci make the case that PU potentially produces a ‘new vocabulary of urbanization through the construction of an ex-centric perspective that dislocates the focus of analysis from its conventional center: the city’ (2018: 512).

Yet, ‘most research [in UPE] while recognizing the globalized societal relationships with nature that constitute urban life today, and the complex governance processes that regulate them, has looked at individual or comparative case studies, not at the networked matrix itself on which urban-nature relations are made and unmade’ (Keil, 2011a: 716). In a critical commentary on the UPE literature, Angelo and Wachsmuth (2014) warn against a ‘methodological cityism’, which ‘refer[s] to an analytical privileging, isolation and perhaps naturalization of the city in studies of urban processes where the non-city may also be significant’ (p. 20; see Connolly, 2018, for a response). While there is nothing inherently wrong per se with research carried out in cities, they suggest there is a danger to this being the overwhelming norm: ‘An urban studies that is (city) site rather than (urban) process focused thus risks ignoring much of what is distinctive about the contemporary urban world’ (2014: 23). Moreover, McKinnon et al. (2017: 8) write:

In effect, the creation of UPE has, at least to some degree, reinforced the nature-society divide it was attempting to dissolve by reinforcing its analog, the urban-rural divide. Only a few studies in the UPE tradition have worked across this spatial divide – or as some social-ecological scientists might suggest, this gradient – by focusing outside the city proper.

Angelo and Wachsmuth (2014) offer two possible directions for future, more Lefebvrian-focused research. The first: to ‘investigate processes of socionatural transformation that systematically differentiate, within specific regions or at larger scales, city from non-city – in other words, to show how urbanization produces, materially or representationally, spaces understood as urban or rural, or materials understood as natural or social’ (2014: 24). The second: ‘to more rigorously interrogate [urbanization’s] global uneven development, tracing features of the urban world across the planet and integrating those that rarely if ever appear in cities’ (2014: 25). An example is Arboleda’s (2016) work on spaces of extraction, showing how urbanization produces ‘nature’ and ‘space’ well beyond the city through a dialectic of homogenization and fragmentation. Or as Wilson and Jonas (2018: 2) argue, ‘planetary urbanization posits a simultaneity of process, with urbanization best understood by recognizing “temporal flows” of relentless, multi-directional spillages, leakages, causal criss-crosses, and trans-boundary processural connections’. Keil (2018a) likewise encourages a Lefebvrian reaffirmation, identifying neoliberalization and climate change as the forces currently providing the conditions for planetary urbanization (p. 7). He adds, however, that to avoid the very present ‘danger of becoming a vacuous shell for academic debate’, the PU thesis ‘must be politicized again and linked to its revolutionary origins’ (Keil, 2018a: 1591). Viewing the PU thesis as the ‘outcome of half a century of urban struggles’, Keil points to feminist and postcolonial concerns about totalization and universality, but in particular to activist and liberationist concerns from which he expects generative impacts on theorizing (Keil, 2018a: 1591).

Indeed, feminist geographers have offered strident critiques of the planetary urbanization thesis (Buckley and Strauss, 2016; Oswin, 2016; Derickson, 2017; Butcher and Maclean, 2018; McLean, 2018; Peake et al., 2018). For Derickson (2017) planetary urbanization does not appear to be interested in becoming a situated theory; instead it relies on what Donna Haraway (1988) describes as a ‘god trick’ that reproduces a ‘conquering gaze from nowhere’. In other words, while Derickson (2017: 558) shares planetary urbanization’s ‘interest in and concern with the relational and hybrid nature of social relations and their interconnectedness, and a concomitant rejection of the kind of dualisms like urban/non-urban…if these findings are to be effectively political, there are important implications for the production of knowledge’. Oswin (2016) adds the call to ‘queer’ our thinking of the planetary urbanization lens, arguing that the concept can be too comprehensive and violent to other critical urban approaches. In their critical engagements, and with reminders of Lefebvre’s own interest in differences and the everyday, these scholars have affirmed ‘epistemic plurality’ (Buckley and Strauss, 2016), ‘chaotic research pathways’ (McLean, 2018) and ‘other fields of vision’ (Peake et al., 2018). A number of researchers have shown the value of considering the everyday lives of a variety of subjects (Loftus, 2012; Ruddick et al., 2018; Schmid, 2018). As one way forward, Loftus (2018a) renegotiates and transcends the ‘grounded-planetary’ dichotomy, suggesting the two as mutually constitutive, and promotes ‘a philosophy of praxis that begins from lived practices’ (p. 94). Thus, not only is there a need for a Lefebvrian redirection, but a *situated* UPE at that, taking to heart the empirical, theoretical, and methodological insights of feminist and Global South scholarship.

### 2 The call for a situated UPE

The call for situated UPE scholarship mobilizes a Global South perspective as a tool for conceptual and empirical reorientation, rather than simply as an afterthought. This direction enriches the field with new research methods, theoretical framings and practices from the Global South, thus provincializing north-centered UPE debates (Lawhon et al., 2014, 2016; Loftus 2019a Goldfischer et al., 2019). Such scholarship has suggested giving more attention to everyday practices (Loftus, 2012), a more nuanced examination of power as diffused and relational (Lawhon, 2012; Lawhon et al., 2014), and an emphasis on race, gender and location (Njeru, 2006; Truelove, 2011, Loftus, 2019b). Furthermore, the importance of conceptualizing environmental justice issues beyond the usual North–South divide (Ranganathan and Balazs, 2015; see Keil, forthcoming, for an extension of this argument) is only growing as extended urban systems are now being prepared for the climate emergency through global systems of financing, knowledge and engineering (Goh, 2019).

One particularly fruitful focus has been infrastructure, including the production of networked infrastructures beyond the city (Cowen, 2019; Filion and Pulver, 2019; Van Neste, 2019) and the everyday practices related to infrastructure use and delivery (Bhan, 2019; McFarlane and Silver, 2017; Silver, 2014; Simone, 2004). The engagement with infrastructures has always been a critical component of UPE (Kaika and Swyngedouw, 2000; Marvin and Graham, 2001; Young and Keil, 2005), but the call for a situated UPE is in dialogue with the recent ‘infrastructural turn’ in urban studies (Graham, 2010). Lawhon et al. (2018), in a critical response to the idealization of universal, uniform infrastructure by urban theory of the Global North, propose ‘heterogenous infrastructure configurations’ as an analytical lens that, amongst other things, troubles the formal/informal binary by directing research towards ‘the conditions under which particular socio-technical artefacts work, for whom they work, and what it means for infrastructure to work’ (p. 730). Doshi (2017: 125) reminds us that ‘the body is [often] mobilized in conceptualisations of cities and infrastructure while material embodiment remains under-studied and disparately theorized’. Drawing on research in the Global South, she offers five propositions: ‘attention to [embodied] metabolism, social reproduction, intersectionality and articulation, emotion and affect, and political subjectivity’. Similarly, Holifield and Schuelke (2015) call for incorporating the aesthetic mobilization of desires into UPE analyses of process and disruption.

Along with perspectives from the Global South, the call for a situated UPE, in our view, should also include indigenous political ecologies, theories and practices of decolonization, as well as abolitionist political ecologies (Heynen, 2016, 2018a). Indigenous political ecologies are especially relevant in settler colonial societies – such as Australia, Canada and the United States, where suburbanization has been prominent, and where the clash between suburbanization as a way of life and traditional ways of living on the land has been most pronounced (Maginn and Keil, 2019; Middleton, 2015; Veracini, 2012). This extends not just to suburbs or peripheries as places but also as sites and products of relational connectivities. As Kipfer (2018: 474) has shown for the case of pipeline politics in Canada – so central to the continuation of the suburban project in the country and internationally – ecological thinking around extended urbanization cannot do ‘without resorting to…approaches that help us understand the settler-colonial aspects of Canadian urban history and grasp the inter-national dimensions of Indigenous politics’ (see also Hern and Johal, 2018; Pickerill, 2018). Simpson and Bagelman (2018) argue that in occupied British Colombia while a ‘colonial socionatural order’ has been imposed on millennia-old (indigenous) Lekwungen socioecologies, these have never been completely erased, such that the production of nature proceeds through the ongoing interplay of colonization and resistance. A similar call for more emphasis on de-centralizing, ‘counter-hegemonic’ processes comes from Gururani and Vandergeest (2014), who suggest a change in our focus towards ecological knowledge produced by local actors. As Schulz (2017) makes clear, decolonization is not only about recognizing material processes of appropriation and subjugation but also hierarchies of knowing and being that structure research practices: ‘The careful building of a pluriversal dialogue that is neither embedded in culturalism nor absolute particularism, but in the realization that multiple loci of enunciation coexist and are entangled through the coloniality of knowledge, being and power, will thus be the major task that lies ahead for a decolonial-ecological critique in and of the Anthropocene’ (p. 139).

### 3 Addressing the rift between developments in urban policy/politics and developments in the UPE academic debate

Whilst academic debate moves beyond privileging cities as objects of inquiry, cities are increasingly becoming the preferred sites of policy and governance experiments attempting to address climate change: from the UN’s Urban Agenda to circular economies and smart cities experiments, cities are now expected (in policy rhetoric) ‘to save the planet’ (Kaika, 2017; Angelo and Wachsmuth, forthcoming).^2^ Increased attention to cities in policy-making is also reflected in experiments with ‘translocal’ responses (Bulkeley et al., 2014), ‘climate change experiments’ (Broto and Bulkeley, 2013), ‘municipal voluntarism’ (Bulkeley and Betsill, 2013), the changing role of the state (Loftus, 2018b), and a proliferating number of ‘urban laboratories’ across the world (Turner and Kaplan, 2018: 7). Theorizing such governance practices is central in contemporary UPE literature, particularly in the context of neoliberal reorganizations and shifting discourses and practices of urban sustainability, circularity, and resilience (Leitner et al., 2018; Gabriel, 2014; Lynn, 2017).

These debates strengthen the original UPE focus on governance issues. For instance, Cohen and Bakker (2014) investigate how environmental governance is being rescaled through ecological concepts, like bioregions, and suggest that this is a depoliticizing move. They theorize the eco-scalar fix: ‘a process of rescaling and reorganizing governance as a strategy of either internalizing or externalizing socio-environmental externalities, or both, and thereby displacing conflicts and crises, often through the construction of (purportedly “natural”) ecological scales, which simultaneously depoliticize and repoliticize governance’ (2014: 132). Similarly, the Ontario greenbelt has been interpreted as a scalar fix to unlock existing urban-suburban policy conundrums in the Toronto region – in this case to the benefit of the protected greenspace on the suburban and rural fringe and on behalf of growth control measures leading to intensification in related growth centers off the greenbelt (Macdonald and Keil, 2012). Amuzu (2018) articulates UPE with environmental justice in looking at the governance of e-waste. The financialization of risk and green infrastructures or ‘greenfrastructures’ (especially in the urban periphery) is the concern of a growing number of scholars (Christophers, 2018; see also Bryant, 2018; Macdonald and Lynch, 2019; Ouma et al., 2018; see also Harker, 2017, on debt; Loftus et al., 2019). Rice (2014) contributes an investigation of climate change through carbon governance that emphasizes individual behavior instead of attending to carbon intensive development. Mee et al. (2014) construct a UPE of housing through the lens of water while Edwards and Bulkeley (2017, 2018) research ‘climate changed housing as infrastructure’, arguing: ‘climate change reconfigures the circulations of the city in ways that allow both the state and capital to reach further into the home. It does so by transforming who is governing housing, how housing is being governed, and whose housing stands to benefit’ (2017: 1128). In other words, ‘there is no such thing as an unsustainable city in general, but rather there are a series of urban and environmental processes that negatively affect some social groups while benefiting others’ (Heynen et al., 2006: 10). Speaking from a UPE standpoint, Kaika (2017: 91) demonstrates the problem of using resilience uncritically in current literature and policy by criticizing the idea that nature can be ‘injected’ into cities through parks or green roofs. Consequently, she proposes: ‘If we took this statement seriously, we would need to focus instead on identifying the actors and processes that produce the *need* to build resilience in the first place. And we would try to change these factors instead’ (p. 95). Her approach can inform issues of urban design, especially when analyzing the sustainability of ‘cities of the future’, since such analyses are often lacking a deeper probing of the politics and history of environmental challenges (Glazebrook and Newman, 2018). Similarly the non-human domain studies are dominated by positivist science that obscures its Cartesian ideology (Cutts and Minn, 2018).

Nonetheless, the rift over prioritizing (or not) the urban between academic debate and policy/governance practice is also reflected in the UPE literature. UPE literature that is more concerned with questions of policy and governance is less (or not) concerned with problematizing or further engaging with theorizations of urbanization in relation to UPE and questions of environmental governance, and vice versa. Accordingly, in this paper we stress the importance for UPE to anchor itself both on problematizing (sub)urbanization processes and governance questions.

### 4 Rethinking ‘invading’ species: From soil, water and air, to concrete and bacteria

A discussion about inside and outside, core and periphery, the urban and the ex-urban cannot ignore the more-than-human elements involved in the production of space. Expanding common UPE concerns of commodification, circulation, and metabolism to encompass animals, Barua (2016, 2017, 2019) has shown how lively commodities and nonhuman work are part of urbanization processes. Barua and Sinha (2019) have done interesting work on ‘animating the urban’, asking ‘how commodification or metabolisation affects and alters the sentient experience of animals’ (p. 1164; see also Barua, 2014). Gandy has likewise recently considered the intersections of urbanization and nonhuman species (2019), as well as biodiversity more broadly (2016). The more-than-human also seems to be of particular relevance as geographical concepts of ecologies are taking on board explicitly ‘volumetric’ perspectives (Graham, 2016). Still, an interest in more-than-human UPE is yet to benefit from in-depth cross-fertilization and engagement with STS, landscape ecology, or the work of Tsing (1993), De la Bellacasa (2017) and the latest work of Haraway (2016) that cross disciplinary and sub-disciplinary boundaries and disrupt the categories of center/periphery but also of human/more-than-human.

Related to extended urbanization is work on ‘the spread of “invading species”‘, which Wu and Hobbs (2002: 358) refer to as an ‘increasingly important ecological and economic problem’ – a statement that could just as easily refer to our own species and invasions of various kinds. After all, the authors call for ‘incorporating humans’ and their ‘perceptions, value systems, cultural traditions, and socioeconomic activities’ into landscape ecology (Wu and Hobbs, 2002: 364). There have been several attempts since to integrate the analysis of the physical landscape with human activity (Cumming, 2011) but, by mainly focusing on issues of sustainability and especially ‘resilience’, the analysis often misses the mark by taking a de-politicized perspective (Ahern, 2013; Lovell and Taylor, 2013). Landscape ecology literature largely reproduces the dichotomy of ‘urban’ and ‘nature’ (Jennings et al., 2017; Wu, 2013) and such studies even go as far as suggesting that ‘a small set of landscape metrics is able to capture the main spatiotemporal signatures of urbanization’ (Wu et al., 2011: 7).

Non-human life isn’t the only more-than-human consideration in need of attention. Marull et al. (2010: 498) argue that ‘the process of urban sprawl provides the extreme opposite example [of stability brought through a heterogeneous space-time model], since it always seeks to increase its economic competitiveness by increasing the entropy spread to periphery environments’, with the increased production of CO_2_ emissions, waste, concrete, electronics, etc. For example, ‘second only to water, concrete is the most consumed material’ in the world (Gagg, 2014; see also Harvey, 2018: 177), and capitalism’s addiction to concrete goes hand-in-hand with suburbanization, with China, India, the US and Turkey leading the way (Keil, 2018d). In the same way as water provision in cities, or the disruption thereof, illustrates the messy continuity of ‘city’ and ‘nature’ (Kaika, 2005), suburbanization through concretization is a violent, fetishized process of unabated, seemingly immortal expansion (on water’s political ecology see also Swyngedouw et al., 2002). Contemporary construction with concrete, however, has serious environmental issues due to the CO_2_ emissions from concrete’s production (Naik, 2008, DeJong et al., 2010), since producing one ton of cement releases almost as much CO_2_ while the growth rate of cement-related CO_2_ emissions is constantly rising (Chang et al., 2016). There are several serious (environmental) effects that the widespread use of cement causes: soil contamination, water runoff, lung disease from dust. Even papers seemingly exclusive to analyzing soil improvement begin with an immediate emphasis on concretized (sub)urbanization (DeJong et al., 2010: 197). Chang, Im and Cho (2016) propose to look for solutions in biopolymers when addressing the issues of carbon emissions due to the extended use of cement, while bacteria are seen as the new method for concrete to ‘self-heal’ in a process called bacteria-based calcium carbonate precipitation (Wang et al., 2014) and bacteria-induced enzymes are regarded as saviors even against plastic pollution. Instead of asking what underlies such planetary threats, some seek ‘real solutions’ by turning towards ‘the scientific community who ultimately created these “wonder-materials”…to use all the technology at their disposal’ (Gabbatiss, 2018).

The politics of ecology become especially discerning when related to something as fundamental as air and oxygen (reminiscent of the genocidal weaponization of air in the First and Second World Wars). Nowadays the politics of air are becoming increasingly instrumental in oppressive policing of the body and making air an ‘integral part of sovereign power’, as Nieuwenhuis (2018: 90) argues through the case of gassing events during protests globally (Nieuwenhuis, 2016). Gandy (2017) situates urban air through an ontological discussion on ‘urban atmospheres’ and ‘affect’: the (uneven and unequal) geography of air reminds us how ‘air spaces have been constituted in part by the racialized and classed bodies that live, work, and play in them’ (Choy, 2011, cited in Gandy, 2017: 364). While urban areas are generally positioned as sources of heat and pollution that harmfully diffuse to less urbanized areas (Graham, 2015: 196), the movement of air has little concern for such categories as it crosses bodily and territorial boundaries with troubling nonchalance. Nieuwenhuis (2018: 91) proposes an alternative decolonial reconnection of nature and society by ‘seeing the “right to life” not as a hierarchical relationship that originates from a metaphysical authority of human law over “nature” but as recognition for our always already atmospheric being-together-with humans and more-than humans’.

## III Addressing empirical and conceptual challenges: Moving UPE into the suburbs as a fruitful way forward

In the previous sections we discussed UPE in relation to changing/invading material flows across landscapes of extended urbanization. However, the conceptual/theoretical challenges identified above go hand in glove with the need to expand UPE’s methodological and empirical scope. In this section, we address briefly these challenges and suggest a shift of empirical focus on the changing relationships of suburban natures as a possible fruitful expansion and opening of the field.

A key common characteristic of scholarship that moves UPE beyond the city in recent years is a commitment not only to engaging with research beyond urban geography and urban studies, but also a commitment to empirical work that cuts across traditional understandings of the ‘urban’ and goes beyond a focus on the ‘core’. In a series of articles Ekers and Prudham (2015, 2017, 2018) theorize the ‘socio-ecological fix’, which may help us understand landscape transformations without relying on bounded notions of ‘urban’ and ‘rural’ (see also Andreucci et al., 2017). Coplen’s (2018) work on food systems illustrates how following complex supply chains can be a method for research across urban-rural divides (see also Agyeman and McEntee, 2014; Alkon, 2012; Hovorka, 2006). Saguin (2017) explores the production of non-urban ‘hazardscapes’ through urban-rural metabolisms, while Rice and Tyner (2017) offer a compelling UPE of rural mass violence in Cambodia. Gururani (2002) demonstrates how rural women in the Indian Himalayas constitute their identities through everyday practices and calls for a culturally embedded analysis of nature-society relations. Focusing on the Caribbean, Harrison and Popke (2017) begin to theorize ‘island energy metabolism’ and conceptualize the relations between particular materialities of energy sources and islands, and particular territorial, infrastructural, and geopolitical characteristics. A cross-fertilization between UPE and agrarian political economy has also produced significant methodological insight for moving UPE beyond the city. As Karpouzoglou et al. (2018: 491) note: ‘social inequalities arising from land-use change, inequalities in terms of access to safe and clean water, and the management of industrial waste are only some of the pressing issues that will continue to rise in importance and will require a joint endeavor of thinking across UPE and peri-urban scholarship’.

Scholars have also turned the analytical lens of UPE onto suburbanization processes themselves (Keil and Macdonald, 2016; Angelo, 2017; Taylor, 2011). The suburban has traditionally been depicted as the dumping ground of functions or people undesirable to a perceived lively, healthy, desirable core: from factories, nuclear plants, and garbage dumps to retirement homes and revalidation centers. But this perceived relationship between the peripherality of space and the marginality of people has led to a certain blindness in urban literature itself: ‘Few urban political ecologists have paid detailed attention to the views and perspectives of those marginalized in everyday ecologies, and the differences within and among these groups…A new focus on the micro-metabolisms of everyday life beyond the non-human would help urban political ecologists to open up what the urban means to a richness of life that exists within the human species’ (Shillington and Murnaghan, 2016: 1022).

This has changed in recent years, as political ecology research on the spatial periphery often intersects or overlaps with inquiries on social marginalization. Gustafson’s (2015) work in southern Appalachia and Schmidt’s (2017) work on the re-production of wilderness in Houston’s suburbs are cases in point; they both explore how the exurban is produced through local contestations over knowledge and power. Also focusing on practices of marginalization, Batubara et al. (2018) recently explored the politics of flood infrastructure in Jakarta to demonstrate how inequality is reproduced through urbanization processes such as the extraction of cement from the periphery that is utilized to transform the city. Parés et al. (2013: 342) show how the suburbs of Barcelona emerge through a dialectic of capital flows and the materialization of desires for consumption (homes in this case), a kind of intertwined process of morphological suburbanization and new suburban ways of life. Finally, Bruggeman and Dehaene (2017) propose a distributed model of urbanization through a study on the expansion of electricity infrastructures in Belgium across urban and rural spaces.

The very concept of suburbanization is inevitably expanded in these studies. It is understood as a ‘global process’ that exceeds conventional conceptualizations in urban studies but needs to be studied as distinct from (though not unrelated to) planetary urbanization. Tzaninis and Boterman (2018: 58) argue that the transformations of cities and suburbs are not even ‘two sides of the same coin’ but rather resemble a ‘cyclical, non-dichotomous spatio-temporal process’. Keil notes:

As suburbanization becomes the process and suburbanism becomes the way of life of much of the urban revolution, criteria like density, morphology, social composition, etc. must be reevaluated. The notion of suburbanization as dependent on one centre has to be discarded as the form and life of the global suburb take shape through multiple centralizations and decentralizations. (Keil, 2018e: 496)

We argue that in addition to expanding our understanding of marginality, shedding light on socio-environmental processes linked to suburbanization and to new ‘spaces of extended urbanization’ can also go beyond ‘traditional’ research and political discourses on sustainability that focused on urban centers. Given that suburbanization has been ‘sold’ with nature in mind (Keil and Graham, 1998), a fresh political ecological reading of suburbanization is prescient as ‘the suburb’ is still at times understood as both a place of unsustainable sprawl and a space of innovative responses to ecological problems (Alexander and Gleeson, 2018). Consider, for example, the way in which suburbanization conventionally implied that the city moves into, or closer to, its spatial, natural environment. As the example of greenbelts or conservation areas beyond the urban edge shows, nature can be bounded in a process regulating land use. When Berger (2017) speaks about ‘belting future suburbia’ we might add that the belting also works in the other direction: it belts natures as well. A ‘sociology of nature’ for the suburban planet needs to take into account that society now largely takes shape in the sprawling regions of multiple densities that we call postsuburbia. We find at the urban fringe on one hand ancient land rights, rural remnants, agricultural residues, or previously uninhabited bush; on the other hand, we find the sedimented leftovers of industrial society, mines, old factories and other industrial installations that are being reclaimed by open landscape or incorporated into suburban space (Keil, 2018c). While the suburban fringe appears to Berger as ‘a no-man’s land of random, disaggregated and often uncomplementary, informal and uncontrolled land uses’ (2017: 525), we know that both the suburban and the landscape beyond have been structured by generations or millennia of preceding human-nature interactions. To phrase it in these terms – ‘no-man’s land’ – might risk steamrolling over generations of human-non-human societal relationships with nature as well as the indigenous relationships to land that have existed there for a long time.

Sieverts, theorist of the in-between city (2003), has given us an interesting perspective on the future of these lands. He notes that the *Zwischenstadt* may be the historico-geographic terrain on which new forms of ‘rurbanity’ might help sustain life on a planet of 10 billion. This would mean the ‘merging of urban and rural, of cultural and natural characteristics in this urbanization process’ (2017: 3), including an increase in food production, heightened contradictions of industrial agriculture with more diverse forms of cultures in and around cities, and the spread of ‘horizontal metropolises’ that will have to develop ‘their wildnesses, their areas of adventure and recreation, in themselves, as fractal urban landscapes’ (2017: 4). Sieverts ends with a (rhetorical) question: ‘Why should, under the constraint of inclusion into natural metabolisms, the greatest urban transformation in human history that we have sketched here not lead to fascinating forms of an urban-rural continuum, fascinating new urban landscapes?’ (2017: 4; see also Keil, 2020). Sieverts adds that what has appeared rural and urban at the metropolitan fringe is now being redefined in an anthropogenic context. An apparent conversion is taking place where emerging suburbia and postsuburbia abuts a barren nature outside and a fertile nature inside:

compared to the open countryside, the city offers a protected and safe living space. The humans who live in the city do not represent a menace for plant and animal life. On the contrary, city dwellers tend to be environmentalists. Some of these activities, such as urban gardening, tree adoptions and bird nesting aids, or even the keeping of beehives, add to the quality of the biotope infrastructure. (Sieverts, 2018)

As cities grow outward into a landscape of financialized and industrialized monocultural agriculture *à la Blade Runner 2049*, the rich socio-ecological relationships that one would historically have expected to go beyond the suburbs, in the layered landscapes of the countryside, now move to the city itself which, especially in reaction to climate change, takes on certain aspects of ‘organic’ and collective organization. The paper concludes with propositions to increase attention to areas described as ex-urban, peri-urban, and sub-urban, encompassed into a suburban political ecology that can give us a better empirical and conceptual understanding of the production of new spaces of marginality and of new processes leading to environmental hazard.

## IV Conclusion: Towards a situated more-than-urban political ecology

Although Harvey (1996) correctly argued that ‘there is nothing unnatural about New York City’, there is nothing ‘natural’ about it either (Keil, 2003). Despite recent trends of urban ‘gardening’ or ‘agriculture’, cities will never be materially self-sufficient (McKinnon et al., 2017) and will continue depending on the periphery and generally the spaces that provide urbanism with its sustenance through ‘exploitation and exclusion’, as Ruddick’s (2015: 1122) ‘para-sites’ suggest. Hence ‘seeing like a suburb’ can become a new imperative for political ecology (Ekers et al., 2012), and instead of considering airports, oil fields and garbage dumps as ‘non-places’ and seeing them from the inside outwards, we may begin with them and go from the outside inwards. Furthermore, ‘the anthropological machine reveals a discursive framing that structures the organization of the urban, not as a form but as an edge, an orientation, acting as a dividing line that operates both within the interiority of the urban and between the urban and its nonurban other’ (Ruddick, 2015: 1114).

Through developing a more-than-urban political ecology our concerns can include massive production sites, logistics ‘cities’, brutalscapes, deforestation and vast agricultural landscapes, but also suburban residential sites, be it concrete high-rises or picket-fenced homes. And considering how suburbanization has been targeted as an environmental catastrophe, it is not only poetic but imperative to become part of the solution and not the problem. As Loftus (2018) suggests to reconcile the planetary with the everyday, similarly Keil proposes (forthcoming) to focus on ‘the quotidian revolutions in the sub/urban political ecologies of everyday life’ through which we can ‘reconcile seemingly opposing claims between situated UPE and the call for a post-cityist UPE’. Here is where suburbanization (non-central urban expansion) and suburbanism (suburban ways of life) come together as distinct but inter-connected. ‘It is in the sprawl where sustainability, community and the urban have to be found. It is there where we locate and ultimately transgress the frontiers of urban political ecology’ (Keil, 2011b). This begins not with consensus regarding ‘sprawl’ and the unsustainability of suburbs but with acts of ‘dissensus’ as living indicators for tackling socio-environmental inequality (Kaika, 2017; see also Velicu and Kaika, 2017). After all, nowadays some of the most dynamic socio-political changes happen in the periphery (Caldeira, 2013; Hamel and Keil, 2015; Keil, 2013, 2018d; Ranganathan, 2014; Ranganathan and Balacz, 2015; Roy and Crane, 2015).

‘What and who my communities are during one day and how they need to be sustained changes continuously. In order to find my way through those mazes of relationships, I need to start where I am and not in an imaginary place that is either reviled (like sprawl) or celebrated (like the compact city)’ (Keil, 2011b). Valdivia’s (2018) recent work is one such example that intersects periphery, everyday life and fossil capitalism with the embodied ecologies of an oil refinery city in which conditions of social and chemical toxicity characterize everyday life, but also where desires for social justice manifest through optimism and dignity. As noted in the introduction, our call for a more than urban political ecology also aims to engage with calls to situate UPE (Lawhon et al., 2014; Truelove, 2011; Loftus, 2012) and encourage a better focus on southern urbanisms (Lawhon, et al., 2014; Truelove, 2016; Silver, 2017; Zimmer, 2010; Roy, 2009), the diversity of urban environments (Velzeboer et al., 2018), and everyday practices (Truelove, 2011; Loftus, 2012; Birkenholtz, 2010; Simpson and Bagelman, 2018). As Kipfer (2009: 68) suggests: ‘The urban functions as a level of analysis mediating between macro- and micro-levels of reality and possibility. In other words, the urban leads not only to analysis of the macro-realities of the state, capital and empire but also to a differential and dialectical critique of everyday life’.

There is no outside to the more-than-urban continuum (Lerup, 2017; Newell and Cousins, 2015) and ‘we live, indeed, in a world of continuous massive sub/urbanization. There is no escape from it conceptually or materially’ (Keil, 2018b). Focusing on the more-than-urban, therefore, we might find new openings and possibilities for engagement between human and more-than-human worlds. Yet, the multiplicity of the urban must guide us away from all-encompassing, perennial ideas of what the urban is and what it may entail (i.e. as the anthropocenic approaches imply) (Ruddick, 2015). At its center (and its periphery), the question of the urban condition is a political question that we cannot afford to avoid.

## Notes

1. We are grateful to one of the reviewers who urged us to make this point part of our argument. Some of the ideas and even phrasing in this paragraph are attributed to them and acknowledged here as such.

2. This paper by Angelo and Wachsmuth is the introduction to a special issue with a set of very topical empirical contributions for urban studies. Keil (forthcoming) is a commentary which also reflects on the papers in the special issue.
